# Nitration of tyrosines in complement factor H domains alters its immunological activity and mediates a pathogenic role in age related macular degeneration

**DOI:** 10.18632/oncotarget.14940

**Published:** 2017-02-01

**Authors:** Matthew Krilis, Miao Qi, Michele C. Madigan, Jason W. H. Wong, Mahmoud Abdelatti, Robyn H. Guymer, John Whitelock, Peter McCluskey, Peng Zhang, Jian Qi, Alex P. Hunyor, Steven A. Krilis, Bill Giannakopoulos

**Affiliations:** ^1^ Save Sight Institute, University of Sydney and Sydney Eye Hospital, Sydney, NSW, Australia; ^2^ Department of Infectious Diseases, Immunology and Sexual Health and Department of Medicine, St George Hospital, University of New South Wales, Sydney, NSW, Australia; ^3^ School of Optometry and Vision Science, University of New South Wales, Sydney, NSW, Australia; ^4^ Prince of Wales Clinical School, University of New South Wales, Lowy Cancer Research Centre, Sydney, NSW, Australia; ^5^ Centre for Eye Research Australia, Royal Victorian Eye and Ear Hospital, University of Melbourne, Melbourne, Victoria, Australia; ^6^ Graduate School of Biomedical Engineering, University of New South Wales, Sydney, NSW, Australia; ^7^ Department of Cardiothoracic Surgery, Tianjin Medical University General Hospital, Tianjin, China; ^8^ Department of Rheumatology, St George Hospital, Sydney, NSW, Australia

**Keywords:** macular degeneration, complement factor H, nitrosative stress, retina

## Abstract

Nitrosative stress has been implicated in the pathogenesis of age related macular degeneration (AMD). Tyrosine nitration is a unique type of post translational modification that occurs in the setting of inflammation and nitrosative stress. To date, the significance and functional implications of tyrosine nitration of complement factor H (CFH), a key complement regulator in the eye has not been explored, and is examined in this study in the context of AMD pathogenesis.

Sections of eyes from deceased individuals with AMD (n = 5) demonstrated the presence of immunoreactive nitrotyrosine CFH. We purified nitrated CFH from retinae from 2 AMD patients. Mass spectrometry of CFH isolated from AMD eyes revealed nitrated residues in domains critical for binding to heparan sulphate glycosaminoglycans (GAGs), lipid peroxidation by-products and complement (C) 3b.

Functional studies revealed that nitrated CFH did not bind to lipid peroxidation products, nor to the GAG of perlecan nor to C3b. There was loss of cofactor activity for Factor I mediated cleavage of C3b with nitrated CFH compared to non-nitrated CFH. CFH inhibits, but nitrated CFH significantly potentiates, the secretion of the pro-inflammatory and angiogenic cytokine IL-8 from monocytes that have been stimulated with lipid peroxidation by-products. AMD patients (n = 30) and controls (n = 30) were used to measure plasma nitrated CFH using a novel ELISA. AMD patients had significantly elevated nitrated CFH levels compared to controls (p = 0.0117). These findings strongly suggest that nitrated CFH contributes to AMD progression, and is a target for therapeutic intervention.

## INTRODUCTION

Age-related macular degeneration (AMD) is a leading cause of irreversible vision loss in people aged over 50 years in developed countries [1].

Multiple lines of evidence suggest that dysregulation of the alternate complement system is an important contributor to AMD pathogenesis [2]. Furthermore, 30-50% of genetic susceptibility to AMD is conferred most strongly by the complement factor H (CFH) Y402H polymorphism, resulting in a tyrosine(Y)-to-histidine(H) substitution at amino acid position 402 within the CFH protein [3–6]. CFH is a major negative regulator of the alternate complement pathway [7]. The alternate complement system is triggered when C3 is converted to C3b. C3b forms the C3 convertase complex that through a positive feedback loop amplifies C3b generation, which then goes on to form part of the C5 convertase complex, ultimately leading to the formation of the complement membrane attack complex. C3b is promiscuous in its ability to opsonize not only pathogen surfaces but also healthy cell surfaces, and the latter can lead to co-lateral damage of the host cells unless C3b is promptly inactivated by regulatory proteins such as CFH at these sites [7].

CFH is an abundant plasma protein that is composed of 20 complement control protein modules or domains. CFH promotes Factor I mediated C3b inactivation by binding C3b predominantly through domains 1-4 at the N-terminus of the CFH molecule [8]. CFH can provide Factor I cofactor activity to inactivate C3b either in the plasma fluid phase or on the surface of cells [8]. CFH can also localize to Bruch's membrane (BM) (a semi-permeable structure that separates the choroidal blood supply from the retina) by binding to glycosaminoglycans (GAGs) such as heparan sulphate [9], a major type being perlecan [10]. CFH domains 6-8 and 19-20 have been shown to play an important role in heparan sulphate binding, resulting in a conformation that allows domains 1-4 of CFH to then interact with C3b [8]. Domains 7 and 20 have also been shown to be important in facilitating CFH binding to malondialdehyde (MDA) and malondialdehyde acetaldehyde (MAA) which are lipid peroxidation byproducts detected in drusen deposits in AMD eyes [11]. CFH binding to MDA/MAA inhibits amplification of the alternate complement system by promoting C3b cleavage to inactive iC3b fragments [11]. Of note the Y402H variant of CFH displays significantly diminished binding to heparan sulphate [9] and to MDA/MAA modified molecules, and concurrent loss in its ability to inactivate C3b [11]. This provides a mechanistic account of how this polymorphism leads to dysregulation of the alternate complement system in AMD.

Tyrosine nitration is a type of oxidative post-translational modification of proteins that occurs as a downstream consequence of peroxynitrite mediated attachment of a NO_2_ moiety to a tyrosine residue within the protein [12]. Peroxynitrite is generated in the setting of excess levels of reactive oxygen species in the presence of nitric oxide or nitric oxide derived metabolites (also termed nitrosative stress) [12]. Tyrosine nitration has been shown to affect extracellular proteins in BM, and the degree of nitration increases with age [13]. The possible relevance of tyrosine nitration to the pathophysiology of the ageing process has previously been emphasized [14]. Whether tyrosine nitration of complement regulatory proteins such as CFH occurs, and if so what are the functional implications of this, has not been explored to date, and is the aim of this study in the context of AMD pathogenesis.

## RESULTS

### Co-localization of CFH and nitrotyrosine immunoreactivity in the choroid and retina of AMD eyes

**Figure 1:**
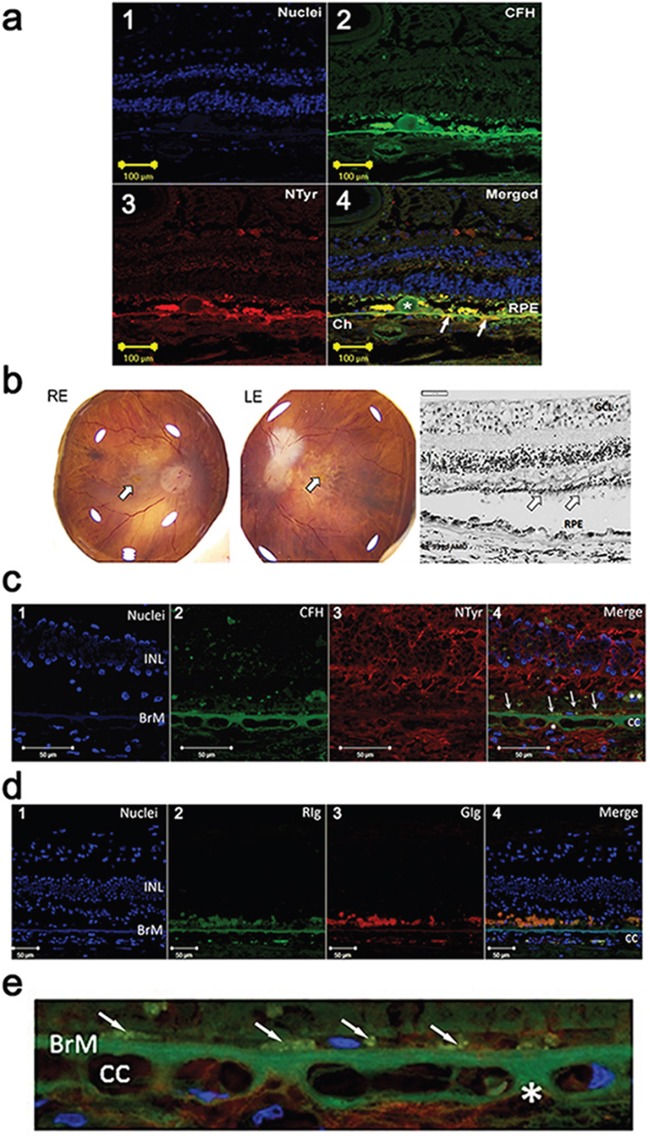
**a**. Immuno-localisation of CFH and nitrotyrosine in the choroid and retina in an early AMD human eye (1-4). (1) Nuclei are counterstained with bisbenzimide (blue). (2) Double immunolabelling for CFH (green) and (3) nitrotyrosine (NTyr) (red) in the retina and choroid from an eye with early AMD (83 year old, female, AREDS Grade 1). Image in 4 is merged and shows colocalisation (yellow) of CFH and NTyr in Drusen (*), RPE, Bruch's membrane and extracellular matrix around the choriocapillaris (Ch). Arrows indicate site of colocalisation. **b**. Right (RE) and left (LE) postmortem eyes (93 yo, dry AMD AREDS grade 4) photographs showing areas of atrophy in the macular region of each eye (arrow – fovea). Representative image of a haematoxylin & eosin stained paraffin section of the retina and choroid from the RE dry AMD lesion, showing disrupted retinal pigment epithelium (RPE) and sub-RPE deposits. Note the thinned outer nuclear layer and remnants of photoreceptor outer segments (arrows) (GCL: ganglion cell layer; INL: inner nuclear layer). **c**. Immuno-localisation of CFH and nitrotyrosine in the choroid and retina in the human eye shown in **b**. (1) Nuclei are counterstained with bisbenzimide (blue). (2) Immunostaining for CFH (green) and (3) NTyr (red). (4) Colocalisation of CFH and NTyr are seen in the BrM (arrows point to area of colocalisation). Small areas of CFH and NTyr co-localisation (merge, *) are seen in the extracellular matrix around the choriocapillaris (cc). Remnants of RPE are visible (**) in this area, and the outer nuclear layer is not visible, associated with outer retinal atrophy. **d**. Immunolabelling with immunoglobulin (Ig) control (1) Nuclei are counterstained with bisbenzimide (blue). (2) Rabbit (RIg) (green) and (3) goat (GIg) (red) shows some non-specific immunolabelling (cc: choroiocapillaris; INL: inner nuclear layer; BrM: Bruch's membrane). **e**. High power image of Figure 1c (4) demonstrating immuno-localisation of CFH (green) and nitrotyrosine (red) in the Bruch's membrane (BrM) / choroiocapillaris region of the dry AMD human eye. Nuclei are counterstained with bisbenzimide (blue). Small areas of CFH and NTyr co-localisation (merge, *) are seen in the extracellular matrix around the choriocapillaris (cc) and in cell remnants just above Bruch's membrane (arrows).

Paraffin sections of AMD eyes (n = 5, age of patients 69, 78, 83, 84 and 93 with Grade 1 to 4 Age-Related Eye Disease Study (AREDS) staging were examined for expression of CFH and nitrotyrosine using single and double immunolabelling and confocal microscopy. A monoclonal antibody that specifically binds to 3-nitrotyrosine was used to detect the latter. We observed areas of colocalisation of CFH and 3-nitrotyrosine immunoreactivity in the extracellular matrix (ECM) surrounding the choriocapillaris, including the choriocapillaris pillars in all specimens; evidence of colocalization also was observed in BM in some specimens. Representative examples from two patients with AMD are shown in Figure 1a (1-4) is from an 83 year old female AREDS Grade 1 and b-e is from a 93 year old female AREDS Grade 4.

### Characterization of a specific monoclonal antibody to nCFH

**Figure 2:**
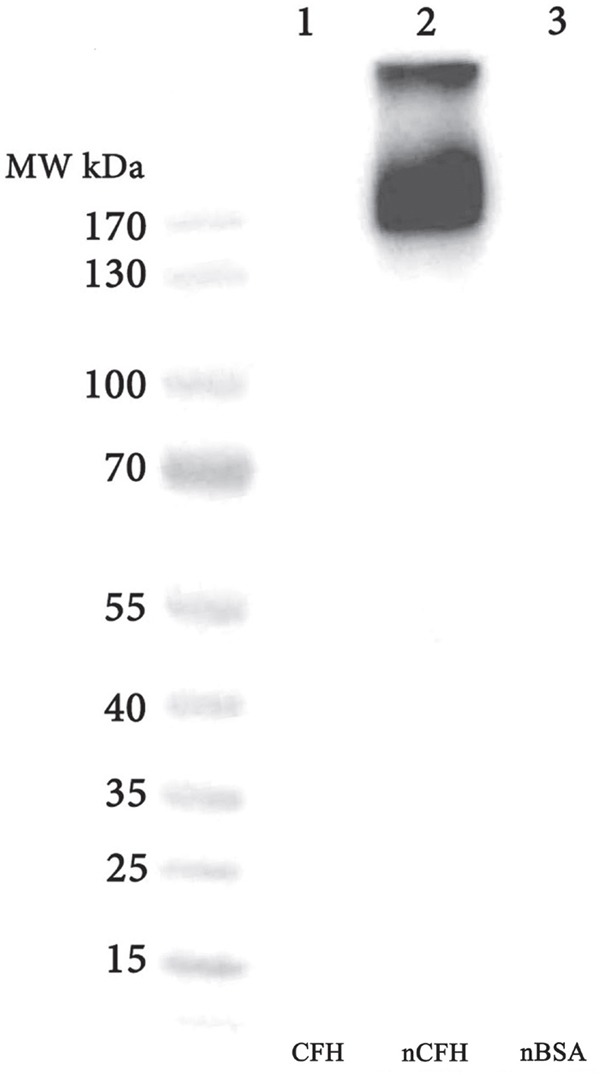
Western blot of CFH and BSA preparations probed with the specific anti-nCFH monoclonal antibody A major immunoreactive band migrating at ∼ 170 kDa and a minor band migrating at >300 kDa (lane 2) was observed with purified CFH that was nitrated *in-vitro*. There was no immunoreactive band detected with CFH (lane 1) and nBSA (lane 3).

On Western blotting the monoclonal anti-nitrotyrosine specific antibody (C166) bound to CFH that was tyrosine nitrated *in vitro* (using the method of heme dependent nitration) [15], but not to nitrated (n)BSA prepared in an identical manner. Furthermore, it did not bind non-nitrated CFH (Figure 2), confirming its restricted specificity for nitrated (n)CFH on Western blotting.

### Identification of nCFH in retinal extract from 2 deceased patients with early and late AMD

**Figure 3:**
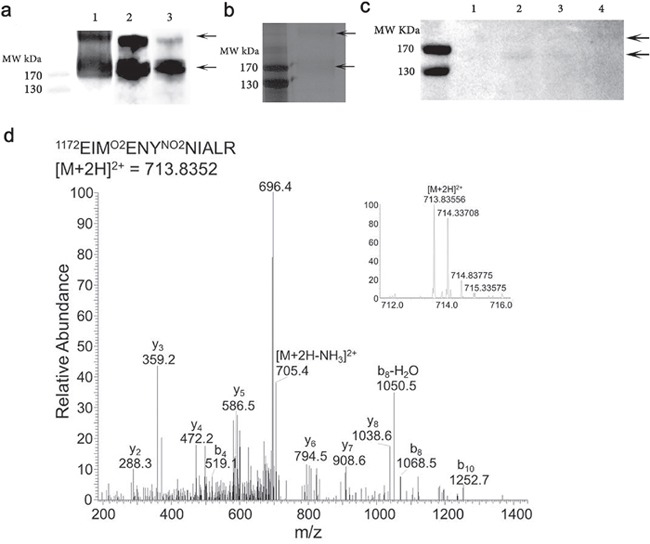
Purification of nCFH from the retina of a deceased individual with AMD (83 year old, female, AREDS Grade 1) **a**. Lane 1 demonstrates a Western blot of whole protein extract from the AMD retina probed with the C166 antibody. Lane 2 affinity purified sample obtained by applying the whole protein extract from the AMD retina to an anti-nCFH monoclonal antibody affinity column probed with the C166 antibody. Lane 3, the affinity purified sample probed with a specific antibody to CFH. **b**. Silver stain of the affinity purified AMD retinal extract. The two arrows indicate the bands that were excised and subjected to tandem mass spectrometry to identify the nitrated residues. **c**. Western blot of whole protein extract from distinct regions of the retina from a patient with AMD (93 year old, female, AREDS Grade 4) probed with the C166 monoclonal antibody.1 = retina without macula, 2 = macula retina (8 mm trephine), 3 = choroid/RPE from below macula (8 mm trephine), 4 = choroid/RPE from rest of eye. **d**. An example tandem mass spectrum showing the detection of NO_2_Y1177 (Domain 20), from the affinity purified sample **(b)**.

One unfixed donor eye (83 year old female AREDS Grade 1, cause of death an intracerebral haemorrhage) with evidence of macula drusen was dissected and the retina including the choroid gently removed and snap frozen in liquid nitrogen and extracted as indicated in methods. Figure 3a shows total protein extract (lane 1). Affinity purified material was isolated from the total protein extract derived from the retina (lane 2). The total protein extract was subjected to affinity purification with the specific monoclonal antibody derived against Ac-KEKKCS (YNO2) TED-NH2 peptide sequence from Apolipoprotein H (designated C166). The total protein extract and eluate were subjected to Western blotting with the anti-nitrotyrosine CFH monoclonal antibody C166 (Figure 3a, lanes 1 and 2 respectively) to confirm that the affinity purification isolated nCFH. Only two immunoreactive bands were identified (lanes 1 and 2) which migrated with identical molecular weights to those observed with immunostaining on Western blot with the C166 monoclonal antibody of native CFH nitrated *in vitro* (Figure 2, lane 2). The affinity purified retinal sample was also probed with a different antibody that binds to non-nitrated CFH demonstrating immunoreactivity at molecular weights identical to those detected with the C166 monoclonal antibody (Figure 3, lane 3). The affinity purified retinal extract was subjected to SDS-PAGE and stained with silver which demonstrated two faint bands (Figure 3b) at the identical molecular weight to those identified on Western Blotting of the total protein and affinity purified extracts (Figure 3a).

A second donor eye (93 year old female AREDS Grade 4, cause of death a myocardial infarction) with end stage dry AMD was dissected and the retina carefully separated from the choroid. The macula was removed using an 8 mm trephine and isolated from the rest of the retina, with further separation of the choroid/retinal pigment epithelium (RPE). The individual samples were extracted and processed as indicated in methods. The extracts from the various locations of the eye were subjected to Western blotting with the monoclonal anti-nCFH antibody C166. The immunostaining demonstrated bands at MW of ∼170kDa and >170 kDa in the macula retina (Figure 3c, lane 2). There was faint staining of the same MW bands in the retina without the macula (Figure 3c, lane 1) and the choroid/RPE from below the macula (Figure 3c, lane 3). There was no staining detected in the choroid/RPE from below the non-macular retina (Figure 3c, lane 4).

Arrows indicate molecular weight of nCFH reactive bands.

### Mass spectrometry identified the CFH tyrosine residues that are nitrated in AMD retina

The two silver stained bands indicated by the arrows in Figure 3b were excised and subjected to tandem mass spectrometry to identify the nitrated residues and the specific proteolytic peptides in which they occur.

**Figure 4:**
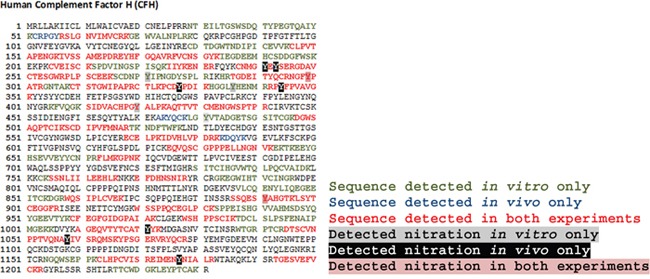
Summary of all CFH nitrated peptides detected *in vitro* and *in vivo* by tandem mass spectrometry Sequence coverage of peptides is shown in different colours.

The mass spectrometry analysis identified CFH as the only protein contained within these 2 bands (with the exception of contaminant proteins including trypsin and keratin), confirming the specificity of the antibody C166 for nitrated CFH. The two immunoreactive bands migrate at a higher molecular weight than non nitrated CFH which could be due to nitration and/or multimer formation. The CFH tyrosine residues that are nitrated in the retina of a deceased individual with AMD, are summarized in Table 1. For peptides containing multiple tyrosine residues, the site of nitration was determined based on the theoretical nitrated peptide tandem mass spectrum with highest ion score as determined by Mascot. An example tandem mass spectrum showing the detection of NO_2_Y1177 in domain 20 of CFH is shown in Figure 3d. Overall, 35% coverage of the CFH derived from the retina was achieved by the mass spectrometry analysis. There was no coverage of the tyrosines in Domain 7 nor for some of the other domains (Figure 4). The amount of CFH protein available for *in vitro* nitration and subsequent analysis was significantly higher than the *in vivo* samples which required multiple purification steps which included homogenisation, lysis, acetone precipitation to remove detergent and affinity purification with the C166 monoclonal antibody affinity column. This means there is significant loss of protein at each step and hence the amount of nCFH for analysis was simply limited for mass spectrometry detection.

**Table 1:** Summary of all CFH nitrated peptides detected by tandem mass spectrometry

Peptide	Nitrated residue	CFH Domain	*In vivo*	*In vitro*
CNMG**Y**E**Y**SER	241, 243	4	x	x
SCDNP**Y**IPNGDYSPLR	271	5		x
NGF**Y**PATR	299	5	x	
CTLKPCD**Y**PDIK	327	6	x	x
HGGL**Y**HENM(+16)R	336	6		x
RP**Y**FPVAVGK	344	6	x	x
SIDVACHPG**Y**ALPK	420	7		x
LG**Y**VTADGETSGSITCGK	481	8		x
SSQES**Y**AHGTK	891	15	x	
AGEQVT**Y**TCAT**Y**YK	1021	17	x	x
DTSCVNPPTVQNA**Y**IVSR	1058	18	x	x
EIMEN**Y**NIALR	1177	20	x	x

A nitrated peptide is marked as detected if it was identified by the Mascot algorithm. Location of the nitrated tyrosine is determined based on the theoretical nitrated peptide tandem mass spectrum with the highest Mascot score. The CFH domain is based on the protein annotation from the UniProt database. Tyrosines included were detected at greater than 10% nitration. The Y in bold denotes the nitrated tyrosines.

### Tyrosine residues of CFH that were nitrated *in vitro*

In order to perform *in vitro* functional experiments using nCFH, CFH was nitrated *in vitro* using the method of heme-dependent nitration [15] and we confirmed the nitration of specific tyrosine residues using mass spectrometry as described above. Overall 59% coverage of the CFH nitrated *in vitro* was achieved by the mass spectrometry analysis (Figure 4). Table 1 summarises the nitrated residues (>10% nitration, quantified by relative ion intensity of nitrated peptide versus the unmodified peptide) and their domain location in the *in vivo* and *in vitro* derived CFH.

### CFH but not nCFH binds MDA-LDL and MAA-BSA

**Figure 5:**
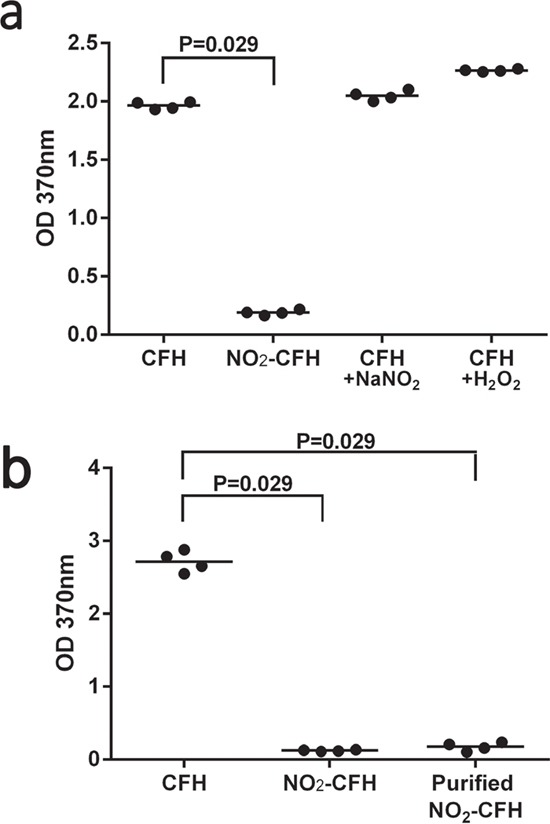
**a**. Binding of MDA-LDL to CFH preparations – binding of MDA-LDL to CFH, nCFH, CFH plus NaNO_2_ or CFH plus H_2_O_2_ was assessed by ELISA. Bound MDA-LDL was detected using a specific anti-MDA antibody. There was significant binding to CFH, CFH plus NaNO_2_ or H_2_O_2_ but not to nCFH. **b**. Binding of biotinylated MAA-BSA to CFH preparations – binding of biotinylated MAA-BSA to CFH, nCFH or purified nCFH was assessed by ELISA. Bound biotinylated MAA-BSA was detected using streptavidin HRP. NO_2_-CFH = nCFH.

We were interested to know whether nitration of CFH modified its binding to MDA or MAA modified proteins and lipoproteins. Figure 5a demonstrates the degree of binding of MDA-low density lipoprotein (LDL) to CFH, nCFH, CFH plus NaNO_2_ or CFH plus H_2_O_2_. There was specific binding to CFH and the CFH controls treated with NaNO_2_ or H_2_O_2_ but only background binding to nCFH. Figure 5b demonstrates the degree of binding of biotinylated MAA-BSA to CFH, nCFH (with the nitrating mixture) or purified nCFH (nitrating mixture removed). There was specific binding to CFH but only background binding to nCFH or purified nCFH. There was no binding of biotinylated BSA to CFH (<0.1), nCFH (<0.1) or purified nCFH (<0.1) at OD = 370 nm.

### CFH but not nCFH binds the heparan sulphate perlecan

**Figure 6:**
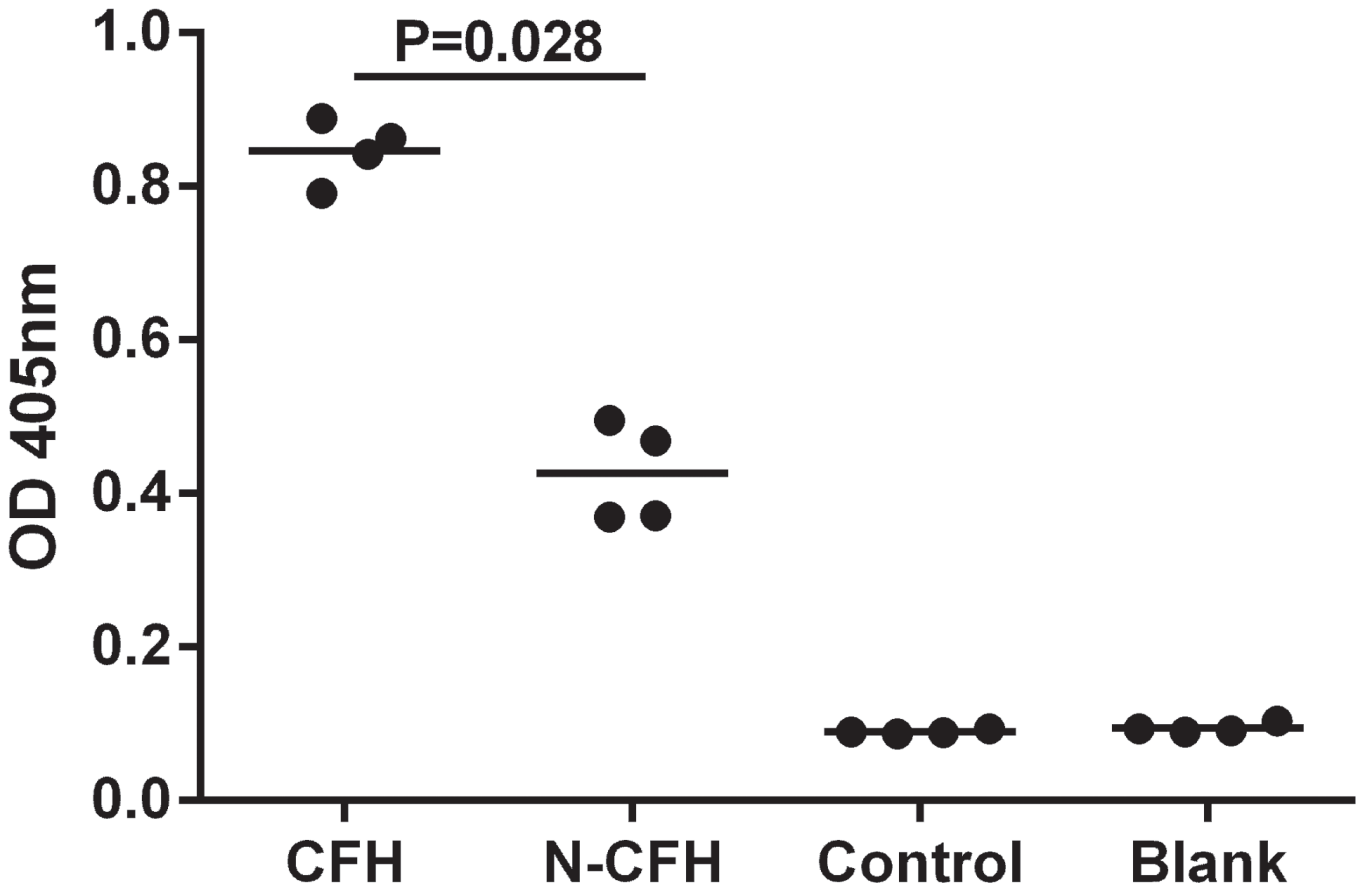
Binding of CFH to immobilized perlecan was assessed by ELISA Bound CFH was detected by an anti-CFH antibody. There was significant binding to perlecan by CFH but not nCFH. The control contained the nitration mixture alone.

Binding of CFH to immobilized perlecan was assessed by ELISA with bound CFH being detected by an anti-CFH antibody. Significant amounts of both CFH and nCFH bound to the basement membrane heparan sulphate proteoglycan, perlecan when compared to control wells. Interestingly, the amount of binding of nCFH compared to CFH was significantly reduced suggesting that the nitration modification of some of the tyrosines is important in the binding (Figure 6). The control contained the nitration mixture alone.

### CFH but not nCFH binds to C3b

CFH bound to immobilised C3b (mean OD = 1.3). In contrast nCFH had significantly less binding (mean OD = 0.31, n = 3, p = 0.024). Treatment of CFH with the nitration mixture controls Cytochrome C (mean OD = 1.2), H_2_O_2_ (mean OD = 1.1) or NaNO_2_ (mean OD = 1.2) did not affect binding at OD = 405 nm.

### CFH mediates cleavage of C3b, which is abolished upon nitration of CFH

**Figure 7:**
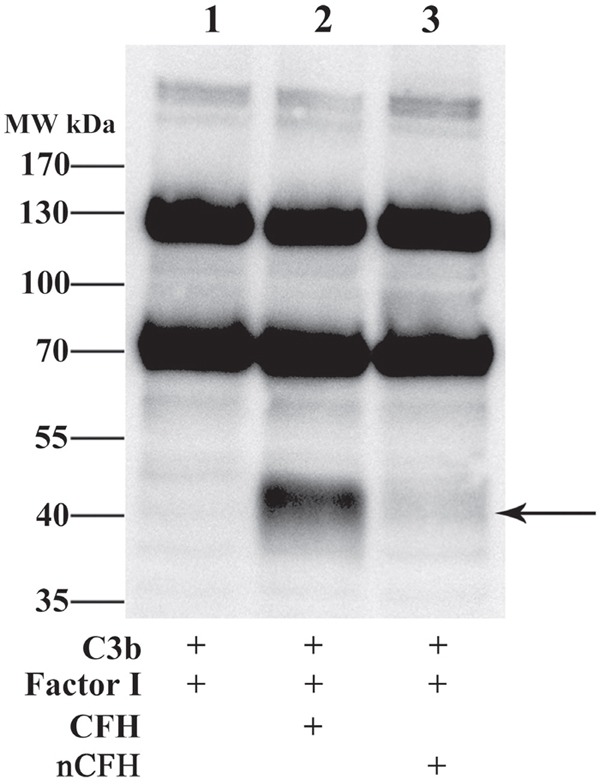
CFH but not nCFH cleaves C3b - Cleavage of C3b by CFH and nCFH was assessed using Western blot C3b was incubated with Factor I and CFH or nCFH and assayed for degradation products. In the presence of CFH, C3b was cleaved by Factor I to a fragment with a mobility at approximately 43 kDa detected with an anti-C3 antiserum (lane 2) whereas in the presence of nCFH, C3b was not degraded by Factor I (lane 3). Arrow indicates degradation product.

CFH provided cofactor activity for Factor I to cleave C3b with an immunoreactive band migrating at approximately 43 kDa, Figure 7, lane 2 indicated by the arrow. However, nitration of CFH abolished the cofactor activity of CFH for cleavage of C3b by factor I (Figure 7, lane 3).

### CFH inhibits, but nCFH significantly potentiates IL-8 release from MAA-BSA stimulated human monocytes

**Figure 8:**
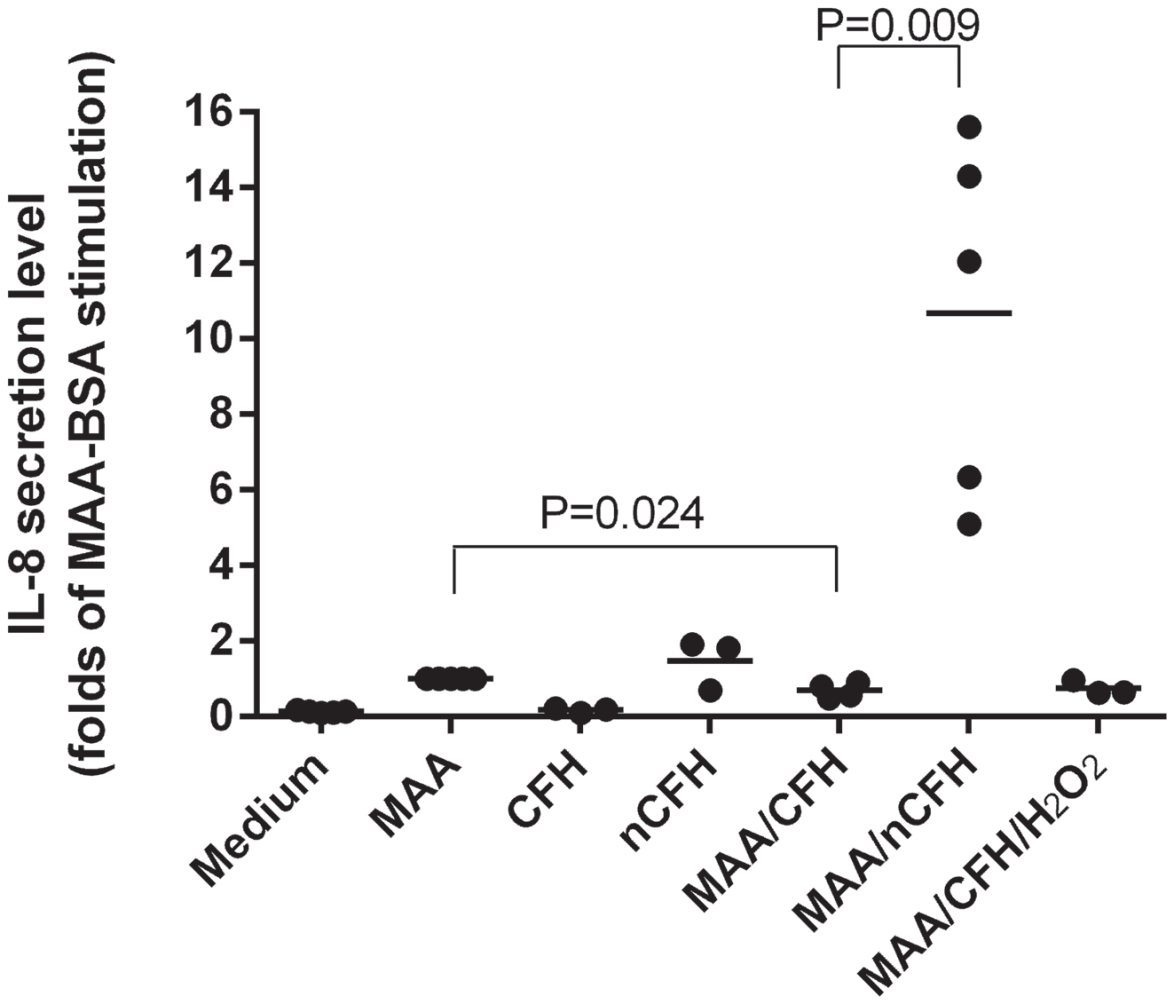
CFH inhibits whereas nCFH potentiates IL-8 secretion from human monocytes stimulated by MAA-BSA IL-8 was secreted by THP-1 cells following stimulation for 18 h with MAA-BSA. The effect of different combinations of CFH and nCFH on IL-8 secretion by THP-1 cells are shown. CFH: complement factor H; nCFH: nitrated CFH; MAA: MAA-BSA; H_2_O_2_: hydrogen peroxide.

CFH but not the nitrated form neutralised the pro-inflammatory effects of MAA-BSA. It has been proposed that the secretion of cytokines, including IL-8, are responsible for the inflammation associated with the pathogenesis of AMD [16]. We were interested to see whether the nitrated form of CFH had any effect on IL-8 production induced by MAA-BSA. MAA-BSA increased IL-8 secretion in THP-1 monocytic cells compared to medium alone, which was significantly inhibited in the presence of CFH (Figure 8). In contrast incubation of THP-1 cells with nCFH induced secretion of IL-8 equal to that of MAA-BSA alone and significantly increased IL-8 secretion when compared to CFH alone (p = 0.03) (Figure 8). In the presence of nCFH, MAA-BSA dramatically increased the secretion of IL-8 up to 11-fold compared to MAA-BSA alone, nCFH alone or MAA-BSA plus CFH. The control consisted of H_2_O_2_ with CFH (Figure 8).

### Plasma levels of nCFH are increased in AMD patients compared to controls

We developed a novel ELISA method for detecting nCFH levels in plasma and compared levels of nCFH in plasma samples from a cross section of AMD patients and controls. AMD patients had statistically significant higher plasma levels of nCFH (4.22 ng/ml ± 8.61, mean ± SD, n = 30) compared to controls (1.73 ng/ml ± 2.97, mean ± SD, n = 30) (p = 0.0109) (see patient and control details in the methods).

## DISCUSSION

In this study we have made the major findings that CFH can exist in a nitrated form *in vivo*, and that nCFH can be detected in retina/choroid of patients with AMD with increased levels in their plasma compared to controls. Mass spectrometry analysis of CFH isolated from an AMD affected retina, using a novel monoclonal antibody that has restricted specificity to CFH nitrotyrosine residues, demonstrated that a number of CFH tyrosine residues that are nitrated in the AMD retina are contained in domains 4, 6 and 20 that serve important functional roles. CFH domain 4 (along with domains 1-3) contributes to binding C3b, providing cofactor activity for Factor I [8]. CFH domains 6 and 20 (along with domains 7/8 and 19) contribute to binding to heparan sulphated GAGs [8], and also permit colocalization of CFH to cell surfaces and onto lipid peroxidation modified debris such as MDA/MAA residues [11]. This allows CFH to limit destructive amplification of the alternate complement system at these sites. We show that the novel nitrotyrosine post-translational modifications we have identified both *in vitro* and *in vivo* in these critical domains impair CFH function in ways that would be expected to amplify the pathogenesis of AMD as discussed below.

Lipid peroxidation byproducts such as MDA/MAA, have been detected in AMD eye samples and can potentiate the activation of the alternate complement system, and elicit a pro-inflammatory cytokine response [11]. IL-8 may also promote new vessel formation [17]. CFH can bind to MDA/MAA residues through domains 7 and/or 20 and limit the activation and amplification of the alternate complement pathway by inactivating C3b to iC3b, and furthermore CFH inhibited MDA/MAA triggered pro-inflammatory cytokine release [11]. Domain deletion mutants of CFH that did not contain either domain 7 or 20 lost approximately 50% of their binding capacity to MDA/MAA [11] respectively. In our study we demonstrate that nitration of tyrosine residues of CFH (Domains 4 and 20) known to play key roles in binding to complement components and MDA/MAA respectively lose the ability to inactivate C3b to iC3b and to bind MDA/MAA residues. Furthermore, rather than merely losing the ability to inhibit MDA/MAA mediated activation of monocytes, nCFH significantly potentiates this proinflammatory effect, suggesting it can amplify the deleterious effects of inflammation in the retina of AMD patients. Cytokines such as IL-8 have been proposed as being relevant in driving AMD lesions [16, 18].

Not all individuals who have the CFHY402H polymorphism develop AMD which suggests that there may be environmental factors that through post-translational modifications further disrupts CFH function, thus allowing AMD to develop. We propose that nitrosative stress may promote AMD progression in individuals who carry the CFHY402H polymorphism by post-translationally modifying the key tyrosine residues of CFH we have identified in the functional domains 4, 6 and 20. The other nitrotyrosine modified CFH residues in domains 15, 17 and 18 we have identified in CFH from AMD retina may also contribute to impairing optimal CFH function and this warrants further investigation.

An important GAG contained in the BM is heparan sulphate [9], which decorates the protein core of heparan sulphate proteoglycans including perlecan, agrin and type XVIII collagen. CFH domains 6-8 and 19-20 play an important role in allowing CFH to bind to heparan sulphate [8], allowing it to localize within BM [19], thus permitting inactivation of C3b in this location. Elderly individuals have been noted to display decreased levels of sulphation in BM [9]. In our study we sought to assess whether tyrosine nitration of native CFH affected its ability to bind perlecan. Nitrated CFH demonstrated significantly lower binding to perlecan compared to non nCFH, suggesting that tyrosine nitration of CFH *in vivo* is likely to significantly inhibit its ability to bind and localize to perlecan within BM and hence limit its capacity to provide sufficient Factor I cofactor activity to inactivate C3b at this location. This may have further implications for AMD pathogenesis as it has been shown that the physiological concentration of CFH competitively inhibits lipoproteins from binding to heparan sulphate residues in the BM hence limiting lipid deposition at this site [20]. Dysregulated lipid deposition has been suggested to be an important contributor to AMD pathogenesis by Curcio and colleagues [21]. Hence we postulate that tyrosine nitration of CFH *in vivo* may potentiate inappropriate lipoprotein build up in BM due to the loss of ability of nCFH to bind heparan sulphate as effectively as non nCFH, leading to loss of ability to competitively inhibit lipoprotein binding to BM.

In this study we were able to demonstrate that quantitation of nCFH in the plasma is feasible, and that elevated levels of nCFH are significantly higher in a cohort of AMD patients compared to controls. This raises the possibility that quantitation of nCFH in plasma may allow for risk stratification in individuals with early AMD, and is an area of research that can be pursued with future well designed, large prospective studies. It will be useful to know whether elevated nCFH levels early in disease predicts greater likelihood of progression to late AMD. In turn elevated nCFH may reflect greater environmental exposure to nitrosative stress, and awareness of such elevated levels may provide impetus for patients to stop smoking cigarettes, modify their diets and perhaps be more compliant with consumption of anti-oxidants with a view to decreasing their risk of developing late AMD. Assessing whether lifestyle modification leads to a decrease in plasma levels of nCFH may also be a useful surrogate marker for adequacy of such preventative strategies in an individual patient, allowing scope for further tailoring of treatment. It will also be useful to know whether elevated nCFH levels in early AMD predicts a greater likelihood of neovascular AMD in view of our findings that nCFH is a potent stimulator of inflammatory cytokines such as IL-8 which is an inducer of angiogenesis [17].

Inhibition of mediators responsible for nitrotyrosine post-translational modifications *in vivo* is a therapeutic avenue for AMD implied by our study. One such target is peroxynitrite formation, an important player in tyrosine nitration pathways. An agent (FeTPPS) that catalytically decomposes peroxynitrite to nitrate has been shown to prevent tyrosine nitration in an *in vivo* diabetic retinopathy model and an *ex vivo* human retina model [22]. As such, agents that remove excessive peroxynitrite accumulation may warrant exploration as a treatment modality in AMD.

## MATERIALS AND METHODS

### Patients

Patient plasma samples were obtained from a well characterised cohort of patients with AMD and controls. The AMD cohort consists of a cross sectional group of 30 patients with either the early stages (n = 17, age 80.6 ± 5.9, 3 male, 14 female) or late (n = 13, age 83.3 ± 6.1, 5 male, 8 female) AMD. Controls (n = 30, age 74.8 ± 9.0, 9 male, 21 female). These patients were well characterised with macular photographs graded for AMD according to the International Classification and Grading System for AMD [23]. Plasma from this cohort has been used in previous studies to examine the association of CRP levels together with the “at risk” genotype of CFH in the prevalence and progression of AMD [24]. Institutional ethics was approved by the Royal Victoria Eye and Ear Hospital Human Research and Ethics Committee (Project #99/3724) and informed consent for patient sampling was obtained from all subjects prior to venepuncture. All methods were carried out in accordance with approved guidelines.

### Reagents

#### Proteins and cell lines

Human CFH, Factor I, C3b, goat anti-C3 antiserum, CFH deficient plasma were purchased from CompTech (Tyler, Texas). Cytochrome C, essentially fatty acid free bovine serum albumin (BSA), anti-mouse alkaline phosphatase (ALP) and anti-rabbit ALP were from Sigma (St. Louis, MO). Perlecan (HSPG2) was purified from conditioned media of human coronary arterial endothelial cells by way of anion exchange and immuno-affinity chromatography as described previously [25].

A biotinylated goat anti-nitrotyrosine antibody was from Abcam (Cambridge, MA). Rabbit polyclonal anti-nitrotyrosine antibody was from Sigma or Merck Millipore (Billerica, MA). Goat anti-CFH antibody was from Millipore. Mouse monoclonal anti-nitrotyrosine specific antibody and mouse anti-CFH antibody were from Thermo Scientific (Rockford, IL). MDA-LDL was purchased from Cell Biolabs, Inc. (San Diego, CA). Anti-MDA HRP was from My BioSource, Inc (San Diego, CA). Streptavidin-HRP, anti-mouse HRP and anti-goat HRP were purchased from Dako (Carpentaria, CA). Isotype control murine IgG2 and rabbit polyclonal IgG were purchased from BD Pharmingen (San Diego, CA). Rabbit anti-goat Alexa 488 or Alexa 594, donkey anti-rabbit Alexa 488 or Alexa 594 were from Molecular Probes, Life Technologies (New York, NY). ELISA for quantification of IL-8 was from BD Pharmingen. The THP-1 monocyte cell line was purchased from American Tissue Culture Collection (ATCC) (Manassas, VA).

### Chemicals

1,1,3,3-Tetramethoxypropane (TMP), Dowex 50W-X4 resin, acetone, trinitrobenzenesulfonic acid (TNBS), acetaldehyde and 2-thiobarbituric acid, 4-(2-hydroxyethyl)-1 piperazineethanesulfonic acid (HEPES), NaNO_2_ (sodium nitrite), hydrogen peroxide (H_2_O_2_) (30% w/w), dithiothreitol (DTT), propidium iodide, iodoacetamide were purchased from Sigma. Pre-cast NuPAGE Novex 4–12% gradient SDS-PAGE gels were purchased from Life Technologies (Madison, WI). Nickel-agarose was purchased from Qiagen (Valencia, CA), MDA was from Cayman Chemical (Ann Arbor, MI), PolyScreen polyvinyldiethylene fluoride (PVDF) transfer membrane and Western blot chemiluminescence reagents from GE Healthcare (Bucks, UK). The biotin labelling kit and trypsin were from Promega (Madison, WI). EZ-Link Sulfo NHS-biotin was from Pierce (Rockford, IL). Super frost Plus slides were from Menzel-Glaser (Saarbruckener, Germany). All other chemicals were reagent grade.

### SDS-PAGE and Western blotting

All samples were resolved on 4–12% gradient SDS-PAGE gels under non-reducing or reducing conditions unless otherwise stated and transferred to PVDF membrane. Unless stated otherwise all primary and secondary antibody reagents were suspended in blocking buffer consisting of Tris-buffered saline – ‘Tween 20′ 0.1% (TBST) / 5% non-fat dried milk. After blocking for 1 h at RT the PVDF membrane was probed at 1 h at RT (unless stated otherwise) with anti–nitrotyrosine, anti-CFH or anti-C3 antibodies. Secondary antibodies were used at final dilutions of 1:2000 for anti-mouse HRP and anti-goat HRP (RT for 1 h). Resultant bands were visualized using chemiluminescence.

Resultant band images were captured using LAS-4000 Image Capture Unit (GE Healthcare, Little Chalfont, UK). Total protein estimations on all samples were performed using the BCA Protein Assay.

### Synthesis of malondialdehyde sodium salt

50 mmols of 1, 1, 3, 3-tetramethoxypropane (TMP) were stirred with 33 ml of the ion-exchange resin Dowex 50W-X4 in 100 ml of ultra pure water for 30 min at RT. The resin was removed by filtration then the filtrate was quickly adjusted to pH 8 by adding 5 M NaOH. The filtrate was extracted three times with equal volume of ethyl acetate and the aqueous layer was dried under vacuum at a temperature below 30°C. The residue was dissolved in a minimum amount of ultra pure water then acetone was carefully added just at the clouding point. The crystalline MDA sodium salt was collected by filtration and recrystallized twice from water-acetone.

### Generation of MAA-BSA

MAA-BSA was generated by incubating 2 mg/mL of BSA with 100 mmol/L solution of MDA (molar mass: 72.0636 g/mol) in the presence of 200 mmol/L acetaldehyde in phosphate buffered saline (PBS) at pH 4.8 for 3.5 h at 37°C in a sealed polypropylene tube in a non-oxidizing atmosphere (argon gas). Unmodified BSA (2 mg/mL) was used as control. Other controls such as MDA- or acetaldehyde-BSA adducts were prepared under the same conditions and at similar concentrations of acetaldehyde or MDA alone when used in the generation of BSA-MAA. Following incubation, unbound MDA and acetaldehyde were removed by extensive dialysis against 3 changes of PBS (2L) for 24 h at 4°C then stored under argon in 50 ml polypropylene tubes at 4°C in the dark.

Unmodified BSA 2 mg/ml was used as control. Modification of BSA with MAA was assessed by the TNBS-test for measuring free amino groups in a protein and by measuring the specific MAA fluorescence spectrofluorometrically with Perkin Elmer Luminescence Spectrometer LS50B (Beaconsfield, England) at excitation wavelength 394 nm and emission wavelength 462 nm.

### Biotinylation of proteins

Biotin labeling of the proteins was performed using EZ-Link SulfoNHS-biotin according to the manufacturer's protocol. The level of biotin incorporation into proteins was determined by HABA assay and yielded an average of 3 molecules of biotin for each molecule of protein.

### Tyrosine nitration of CFH *in vitro*

CFH (1 mg/ml) was incubated in PBS (pH 5.5) at 37°C with NaNO_2_ (sodium nitrite 1 nM), cytochrome C (25 μM) and H_2_O_2_ (3.7 nM). H_2_O_2_ was added in 2 separate aliquots every 15 min over a 30 min period as previously described [26].

### Purification of *in vitro* generated nitrotyrosine CFH

After nitration of CFH, nCFH was purified by electro elution. Briefly, nCFH was separated by SDS-PAGE. The nCFH band identified by Coomassie blue staining was excised and subjected to electro elution using the D-Tube dialyzer. Electrophoresis was performed at 100V over 16 h and electro elution was monitored by the loss of blue color in the gel. The purified nCFH was collected in the D-Tube dialyzer.

### Binding of MDA-LDL to immobilised CFH preparations

CFH, nCFH, CFH plus NaNO_2_ or CFH plus H_2_O_2_ (10 μg/ml) in 0.05 M Carbonate-Bicarbonate (pH 9.6) coating buffer (100 μL) was added to Maxisorp microtitre wells and incubated overnight at 4°C. After blocking with 2% ovalbumin in PBST for 1 h at RT, wells were incubated with MDA-LDL (5 μg/mL) in blocking buffer for 1 h at RT and specifically bound proteins were detected with anti-MDA HRP antibody (1:2000). Optical Density (OD) was measured at 370 nm.

### Binding of biotinylated BSA adducts to immobilized CFH preparations

CFH, nCFH, or purified nCFH (5 μg/ml) in 0.05 M Carbonate-Bicarbonate (pH 9.6) coating buffer (100 μL) was added to Maxisorp microtitre wells and incubated for 1 h at 37°C. After blocking with 2% BSA in PBST for 1 h at RT, wells were incubated with biotinylated MAA-BSA or MAA-BSA or biotinylated BSA (5 μg/mL) as control in PBST (0.25% BSA) for 1 h at RT and specifically bound biotinylated proteins were detected with Streptavidin HRP antibody (1:2000). The OD was measured at 370 nm.

### Binding of CFH and nitrotyrosine CFH to immobilized perlecan

Perlecan (10 μg/ml) in 0.05 M Carbonate-Bicarbonate (pH 9.6) coating buffer (100 μL) was added to Maxisorp microtitre wells and incubated overnight at 4°C after blocking with 2% BSA in PBST for 1 h at RT. Wells were incubated with CFH (10 μg/ml) or nCFH (10 μg/ml) or the nitration mixture in blocking buffer for 1 h at RT and specifically bound proteins were detected with goat anti-CFH (12.5 nM) followed by an anti-goat HRP (1:500). OD was measured at 370 nm.

### ELISA to detect nCFH

A biotin conjugated anti-nitrotyrosine antibody (AbCam) was diluted 1000 fold in 50 mM carbonate bicarbonate buffer and coated on a streptavidin plate for 1 h at RT. After washing 3 times with PBST and blocking with 0.5% ovalbumin in PBST for 1 h at RT, nCFH standard or 100 fold diluted plasma was added in duplicate (100 μl/ well) and incubated for 1 h at 37°C. After washing 3 times with PBST, nCFH bound to the anti-nitrotyrosine antibody was detected with a monoclonal anti-CFH antibody. After washing 3 times with PBST, ALP conjugated goat anti-mouse antibody was added (1:1500 dilution at RT for 1 h) and samples read at 405 nm after addition of appropriate chromogenic substrate. Negative controls were, non nCFH and mouse monoclonal IgG as a control for the primary antibody.

**Figure 9:**
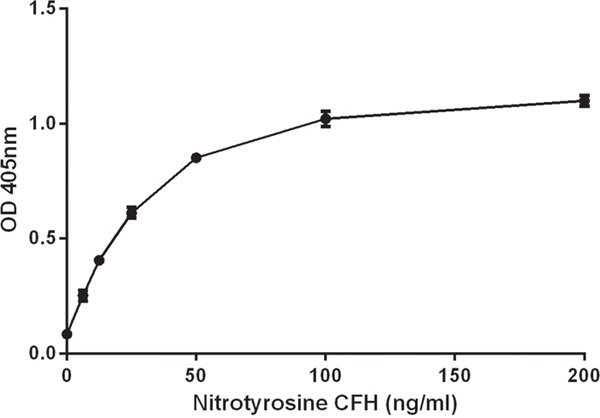
Standard curve for nCFH Purified nCFH at different concentrations was added to CFH deficient plasma to construct the standard curve as described in methods.

An nCFH standard curve was generated for each experiment. Purified nCFH was added to CFH deficient human plasma. The standard curve concentration range for nCFH was between 6.25 ng/ml and 200 ng/ml (Figure 9). CFH deficient plasma without the addition of nCFH had negligible binding and was used as the negative control.

### Determination of coefficient of variation for nitrotyrosine CFH ELISA

Intraplate coefficient of variation (CV) was derived by the equation: Standard Deviation of optical density 1 (OD1) and optical density 2 (OD2) divided by the mean value of OD1 and OD2, multiplied by 100 to give a percentage. OD1 and OD2 are the readings of each point on the standard curve done in duplicate. The interplate CV was derived by dividing the optical density of the 100 ng/ml standard curve sample by the optical density of the 50 ng/ml standard curve sample to obtain a value R for each plate. A total of 7 R values were generated. The interplate CV was derived by the standard deviation divided by the mean of the R values, multiplied by 100 to give a percentage.

### CFH preparations binding to C3b

An ELISA method was used to detect binding of CFH and nCFH to C3b. C3b (5 μg/ml) in 100 μl of 0.05 M Carbonate-Bicarbonate (pH 9.6) coating buffer was added to Maxisorp microtitre wells and incubated for 1 h at 37°C. After blocking with 2% BSA, CFH or nCFH (8 μg/ml) was added to the wells and incubated for 1 h at 37°C. Specifically bound CFH and nCFH was detected with a goat anti-human CFH polyclonal antibody. Controls used were CFH plus H_2_O_2_ and CFH plus NaNO_2_. OD was measured at 405 nm.

### Cofactor activity of CFH and nCFH for cleavage of C3b by Factor I

The cofactor activity of CFH or nCFH or CFH plus H_2_O_2_ or CFH plus NaNO_2_ was measured with an assay that has been described previously [27, 28].

For C3b cleavage, C3b (10 μg/ml), Factor I (0.72 μg/ml), CFH (5 μg/ml) or nCFH (5 μg/ml) were incubated at 37°C for 30 min, then the mixture was separated under reducing conditions by SDS-PAGE and further analyzed by either Western blotting using a goat anti-C3 antiserum (1:2000) or by Coomassie blue staining. The mass spectrometry proteomics data have been deposited to the ProteomeXchange Consortium via the PRIDE [30] partner repository with the dataset identifier PXD005613 and 10.6019/PXD005613.

### Mass spectrometry of *in vitro* generated nitrated CFH

The nitrated native CFH protein was resolved on NuPAGE Novex 4-12% Bis-Tris Gel under non-reducing conditions and stained with colloidal Coomassie. The protein band was excised from the gel, destained, dried and incubated with 100 mM DTT in 25 mM NH_4_HCO_3_ for 1 h at 25°C. The gel slice was washed with NH_4_HCO_3_, dried and incubated with 55 mM iodoacetamide (Sigma) in 25 mM NH_4_HCO_3_. The slices were washed and dried before digestion of the nitrated CFH protein with 20 ng.μL-1 trypsin (Promega) in 25 mM NH_4_HCO_3_ overnight at 30°C.

Peptides were eluted from the slices with 5% formic acid, 50% acetonitrile for 30 min and separated by nano-LC on an Ultimate 3000 HPLC (Dionex) using a fritless nano column (75μ x ∼10cm) containing C18 media (5μ, 200 Å Magic, Michrom). Peptides were eluted using a linear gradient of H_2_O:CH_3_CN (98:2, 0.1% formic acid) to H_2_O:CH_3_CN (64:36, 0.1% formic acid) at 250 nL.min-1 over 60 min. Positive ions were generated by electrospray and analyzed in a LTQ FT Ultra (Thermo Electron) mass spectrometer operated in data dependent MS/MS acquisition mode.

Mass spectral data were searched using Mascot (V2.3, Matrix Science) against the non-redundant database from NCBI. Search parameters were: precursor tolerance 6 ppm and product ion tolerances ± 0.5 Da. Cys-carboxyamidomethyl was selected as a fixed modification and Met-O, Trp-O, Tyr-NO2 and Pyro-Glu/Gln were selected as variable modifications with full tryptic cleavage of up to 2 missed cleavages.

Quantification of the nitrated peptides was performed by calculating the area under the extracted ion chromatogram for the precursor ions (+2 and +3) using the Xcalibur™ Software (Thermo Scientific, v2.2). The mass spectrometry proteomics data have been deposited to the ProteomeXchange Consortium via the PRIDE [29] partner repository with the dataset identifier PXD005613 and 10.6019/PXD005613.

### Human adult eyes

Adult human eyes with age-related macular degeneration (n = 5) (age range 69 to 93 years) were obtained, with consent, from the Lions NSW Eye Bank, and with ethics approval from the University of NSW and University of Sydney Human Research Ethics Committees. All procedures were in accordance with the Declaration of Helsinki. The delay from death to fixation was less than 16 h for all eyes. Following removal of the anterior segment (iris, lens or intraocular lens) and vitreous, the eyecups were rinsed in 0.1M PBS and the retina/choroid examined using a dissecting stereomicroscope and imaged using a digital camera (Leica DFC480) and Photoshop software. Images were used to further classify the eyes where appropriate, using the Age Related Eye Disease Study (AREDS) grading system to assess stages of AMD [30], together with any history of AMD. Human eyes were fixed in 2% paraformaldehyde PBS (pH 7.4) for at least 24 h. Eyes were then dissected and the central region including macular and optic disc, and peripheral nasal and temporal regions paraffin embedded and sections were at 8 μm and collected on Super-Frost Plus slides. Histopathology was assessed in sections stained with Mayer's haematoxylin and eosin (H&E) or Periodic Acid Schiff (PAS) using standard protocols. Stained sections were dehydrated through alcohols and xylenes, then coverslipped in DePeX.

For two postmortem eyes (aged 87 and 93 years), the unfixed retina and choroid/RPE, were gently separated and removed from the eyecup, snap frozen in eppendorf tubes in liquid nitrogen and stored at −80°C for use in protein assays (see above).

### Immunohistochemistry

Sections were dewaxed and rehydrated through alcohols to water. For antigen retrieval, sections were incubated in 0.01M sodium citrate buffer (pH 6) at 95°C for 10 min, cooled to 40°C, and washed with Tris-buffered saline (pH 7.4) and 0.1% Tween-20 (TBST). Sections were incubated at RT in 5% BSA in TBST for 30 min followed by incubation overnight at 4°C in primary antibodies (goat anti-CFH 1:1000, rabbit anti-nitrotyrosine 1:500). Negative controls were treated identically, using non-specific Ig (rabbit or goat Ig) at appropriate concentrations.

After incubation, sections were washed in TBST for 10 min, then incubated with either donkey anti-rabbit Alexa 488 (1:1000) and rabbit anti-goat Alexa 594 (1:1000) for 2 h at RT, and rinsed again in TBS. Cell nuclei were counter-stained with bisbenzimide H33258 (Sigma) (0.1 μg/ml) for 5 min, followed by rinsing in TBS. After a final rinse in TBS, sections were mounted in glycerol, coverslipped and sealed with nail varnish, and dried before microscopy.

### Confocal microscopy

Immunolabelled sections were examined with a Zeiss LSM-5 PASCAL laser scanning confocal microscope system (Carl Zeiss, Germany). Images were collected as single scans using LSM 5 Pascal Version 3.2 SP2 software. Multichannel excitation bleed through was minimized by using fluorochromes with different peak excitations (488 nm and 594 nm respectively). Emission bleed through was minimized by Multi-tracking, where signal crosstalk between neighbouring channels was corrected by performing sequential image capture. The combined images were acquired with an acquisition process using a single excitation /single detection channel. Images were also collected using Z-stacks as appropriate. Images were exported to Adobe Photoshop CS5 for further analysis. Laser excitation levels were constant for all imaging.

### Interleukin-8 secretion by THP-1 human monocytic cell line stimulated with MAA-BSA

Human THP-1 monocytic cells were cultured in RPMI-1640 medium supplemented with 10% FCS. Before stimulation, cells were starved in serum-free RPMI-1640 then incubated with the stimulation medium at a density of 5 × 10^5^ cells/well for 18 h. The stimulation medium contained MAA-BSA (50 μg/ml) and/or combinations of CFH, nitrated CFH, H_2_O_2_, cytochrome C or NaNO_2_ at 100 μg/ml, incubated for 30 min at RT before plating. Cells were removed by centrifugation (1000 rpm, 10 min) and supernatants were assayed for IL-8 by ELISA according to the manufacturer's (Beckton Dickinson) instructions.

### Isolation of whole protein from an AMD affected retina

Total protein was extracted from the liquid nitrogen frozen human retina tissue using the lysis buffer containing 50 mM Tris-HCl, pH 7.4, 0.15 M NaCl, 1 mM EDTA, 0.1% Triton X-100, and 0.1% (wt/vol) SDS with a protease inhibitor cocktail (Sigma). In brief, retinal tissue in 100 μl of lysis buffer was homogenized on ice for 5 min. The supernatant was collected after centrifugation at 5000 g for 10 min. The protein was quantitated using the BCA reagent, and aliquots were stored in −80°C.

### Generation of an anti-nitrotyrosine monoclonal antibody that has restricted specificity to purify nCFH from AMD retina

Hybridoma cells producing a monoclonal antibody (designated C166) was derived to the nitrated peptide AC-KEKKCS(YNO_2_)TED-NH2 sequence from Apolipoprotein H a protein in the same complement control superfamily as CFH using their proprietary technology (Abmart, Berkeley Heights, NJ). For production of purified monoclonal antibodies, hybridoma cells were cultured in culture media DMEM/F12 containing 5% FBS. When cell density reached 1×10^6^/ml, the supernatant was collected after 12 h incubation in serum free medium. The C166 monoclonal antibody in the supernatant was purified using the Montage Antibody Purification Kit (Merck Millipore). The concentration of the purified C166 anti-nCFH monoclonal antibody was measured by Nano-drop 2000c (Thermo Fisher).

### Affinity purification of nCFH from the retina of a deceased AMD patient

The AminoLink® Plus Immobilization Kit (Thermo Scientific) was used to couple the purified anti-nCFH monoclonal antibody. The coupling steps were followed as outlined in the manufacturer's instructions. 1 mg of monoclonal C166 antibody in coupling buffer was added to the AminoLink plus Column and incubated for 4 h at RT. After washing 4 times with the coupling buffer, the column was incubated in coupling buffer plus sodium cyanoborohydride solution for another 4 h at RT. After extensive washing of the column, unbound sites on the column were blocked by the quenching buffer plus the sodium cyanoborohydride solution. After 30 min of incubation and washing 4 times, the column was used to extract affinity purified nCFH from the whole retinal protein extract. The samples containing the retinal protein extract were diluted in PBS buffer and applied to the affinity column. After 1 h incubation at RT, the column was washed with PBS buffer. The bound protein was eluted with elution buffer (0.1M glycine-HCl at pH 2.5-3.0) and the flow through was collected in a tube containing neutralization buffer (1M Tris-HCl at pH 8.5-9.0).

### Statistical analysis

Results were analysed by the Mann-Whitney test. A p value of < 0.05 is taken to signify statistical significance.

## Abbreviations

AMD: age related macular degeneration
CFH: complement factor H
BM: Bruch's membrane
MDA: malondialdehyde
MAA: malondialdehyde acetaldehyde
AREDS: Age Related Eye Disease Study
GAG: glycosaminoglycan
FeTTPPS: 5,10,15,20-tetrakis (4-sulfonatophenyl) porphyrinato iron (III) chloride
HSPG2: heparan sulfate proteoglycan 2
ATCC: American Tissue Culture Collection.
